# *Mustn1* in Skeletal Muscle: A Novel Regulator?

**DOI:** 10.3390/genes15070829

**Published:** 2024-06-23

**Authors:** Charles J. Kim, Michael Hadjiargyrou

**Affiliations:** 1 College of Osteopathic Medicine, New York Institute of Technology, Old Westbury, NY 11568, USA; ckim20@nyit.edu; 2 Department of Biological and Chemical Sciences, New York Institute of Technology, Old Westbury, NY 11568, USA

**Keywords:** *Mustn1*, skeletal muscle, development, repair, knockout, mouse

## Abstract

Skeletal muscle is a complex organ essential for locomotion, posture, and metabolic health. This review explores our current knowledge of *Mustn1*, particularly in the development and function of skeletal muscle. *Mustn1* expression originates from Pax7-positive satellite cells in skeletal muscle, peaks during around the third postnatal month, and is crucial for muscle fiber differentiation, fusion, growth, and regeneration. Clinically, *Mustn1* expression is potentially linked to muscle-wasting conditions such as muscular dystrophies. Studies have illustrated that *Mustn1* responds dynamically to injury and exercise. Notably, ablation of *Mustn1* in skeletal muscle affects a broad spectrum of physiological aspects, including glucose metabolism, grip strength, gait, peak contractile strength, and myofiber composition. This review summarizes our current knowledge of *Mustn1*’s role in skeletal muscle and proposes future research directions, with a goal of elucidating the molecular function of this regulatory gene.

## 1. Skeletal Muscle

Skeletal muscle, a highly complex and specialized tissue, comprises approximately 40% of body mass and represents the most abundant tissue in humans, crucial for enabling locomotion, maintaining postural balance, and ensuring metabolic equilibrium [1]. It consists of intricately structured muscle fibers containing myofibrils predominantly constituted of actin and myosin proteins, whose interaction forms the fundamental contractile unit, the sarcomere. The intricacies of muscle fiber structure pave the way for the exploration of the embryological origin of skeletal muscle, a highly orchestrated developmental process.

**Figure 1:**
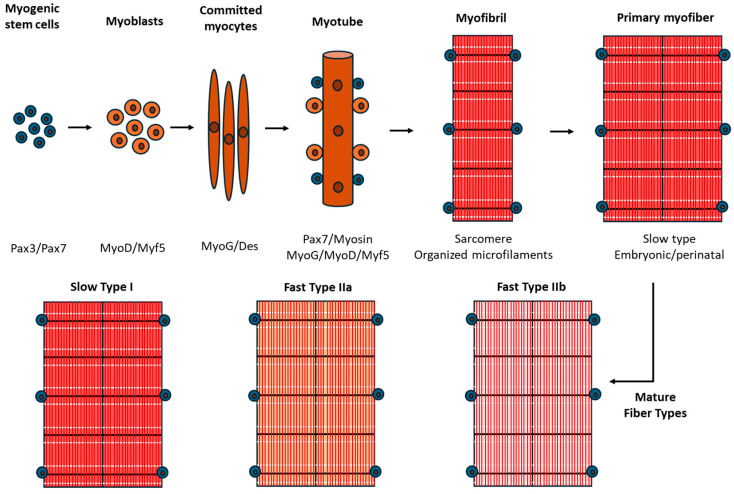
The diagram delineates the progression from stem cells in the paraxial mesoderm to mature myofibers, encapsulating stages of myogenesis during embryonic development in mice. Stem cells expressing Pax3 and Pax7 proceed to asymmetric division and myogenic commitment via MyoD and Myf5 expression. Myoblasts, upon MyoG influence, transition into committed myocytes, expressing Des and myosin. Myocytes undergo fusion, creating myotubes which subsequently differentiate and mature into primary myofibers through the incorporation of myofibrils during primary myogenesis. Secondary myogenesis guides the development of secondary myofibers, influenced by further innervation and myoblast fusion to mature fiber types (slow Type I or fast Type II).

Muscle fibers develop through a complex embryological process wherein precursor cells, or myoblasts, originating from the myotome, undergo a series of migration, fusion, and differentiation to form mature muscle fibers. Multi-nucleated myofibers form the basis of functional skeletal muscle. Specifically, the development of skeletal muscle commences during embryogenesis, when stem cells derived from the paraxial mesoderm (which subsequently forms segmental somites) initiate the expression of markers such as Pax3 and Pax7 (Figure 1). Through asymmetric division, these stem cells sustain the stem cell pool via self-renewal, while simultaneously committing other stem cells to myogenic differentiation via the expression of transcriptional regulators such as MyoD and Myf5. This results in the formation of myoblasts that, under further influence of myogenin (MyoG), transition into committed myocytes and express proteins such as desmin (Des) and myosin heavy chain (MHC). Subsequently, the myocytes fuse, culminating in the formation of myotubes. These myotubes continue to organize myofibrils and finally mature into primary myofibers through a process termed primary myogenesis [2].

In mice, this stage spans embryonic days E11.5 to E14.5, orchestrating the formation of primary myofibers, characteristically slow-twitch Type I fibers, and laying the groundwork for secondary myofiber development. Secondary myogenesis unfolds from E14.5 until birth and encompasses additional innervation and myoblast fusion, ultimately facilitating the emergence of mature myofibers [3] (Figure 1). The specification of fiber type, either slow Type I or fast-twitch Type II, is modulated by genetic factors and motor neuron innervation, collectively influencing muscle strength and functionality [4]. In the case of premature birth, myofiber development does not stop and continues with growth delays and increased risks of potential complications [5,6]. In a study with neonatal rats exposed to high oxygen as an experimental model of preterm birth, the authors observed muscle fiber atrophy, decreased Type I muscle fibers and increased Type IIb fibers, and muscle function impairment. The male gender fast Type II fibers were more affected than the female gender and the slow Type I fibers, with overall effects lasting into adulthood [7].

Postnatal skeletal muscles are terminally differentiated, and thus, growth and repair rely in part on a small population of myogenic precursors, i.e., satellite cells. Postnatal myogenesis, in many respects, recapitulates the intricate processes of embryogenesis, wherein satellite cells are mobilized to undergo sequential phases of activation, migration, proliferation, differentiation, and fusion, either amongst themselves or with existing myofibers, to facilitate repair and hypertrophy of mature skeletal muscles [8]. These cells are typically quiescent under basal conditions, but when activated in the presence of muscle injury or increased physiological demand, they serve as essential mediators for muscle growth, repair, and regeneration. The orchestrated cascade of cellular events during postnatal myogenesis commences with activation, which propels satellite cells to enter the cell cycle, primed for ensuing division and proliferation, rendering them responsive to the demands of compromised muscle tissue.

Prior to activation, satellite cells migrate towards sites of muscle injury or regions necessitating the formation of new muscle fibers. Their migration is precise and pivotal for the alignment of cells to the exact loci where they are needed to ensure the precise repair and formation of muscle fibers. Upon reaching the injury sites, satellite cells undergo extensive proliferation, leading to a reservoir of myoblasts. This phase is indispensable, ensuring a sufficient supply of myoblasts poised to replace damaged muscle fibers or augment muscle mass, thus fulfilling the requirements of muscle repair or hypertrophy. Subsequently, myoblasts undergo differentiation, a transition marked by the end of cell division and the activation of muscle-specific protein expression, signaling their irreversible commitment to the muscle lineage. Ultimately, myoblasts become the mature cells of skeletal muscle.

Further, this transition sets the stage for fusion, the final phase in postnatal myogenesis. Differentiated myoblasts either fuse to form new muscle fibers or integrate with damaged fibers, aiding in their repair. This process is integral to muscle hypertrophy and regeneration, enhancing muscle mass and the restorative capacity of the damaged tissue. Lastly, these highly regulated processes of repair and hypertrophy are fundamental for preserving the integrity and functionality of skeletal muscle. Postnatal myogenesis adapts muscles to augmented workloads and facilitates recovery from injuries, guiding the formation, adaptation, and restoration of skeletal muscle throughout the life of an organism. As such, it truly epitomizes a dynamic system of various phases, each regulated with precision, thus enabling the muscular system to adapt to its form and perform its function, thereby sustaining the demands of the entire musculoskeletal system.

Finally, muscle strength, a critical aspect of tissue function, is primarily determined by the cross-sectional area of the muscle fibers and their ability to generate force. This strength is crucial for maintaining posture, ensuring mobility, and performing various physical activities. The diversity of fiber types corresponds to the various roles of skeletal muscle, with Type I fibers predominantly contributing to endurance and sustained activities, and Type II fibers to power and speed [9,10]. The aforementioned developmental processes not only determine the types of muscle fibers formed but also influence the muscle’s ability to generate force and its adaptability to different physiological demands, ultimately impacting the overall muscle strength. While the ratio of fiber types can adapt over time to the different physiological or pathological demands, genetics is believed to influence approximately 40–50% of this ratio [4].

## 2. *Mustn1*

**Figure 2:**
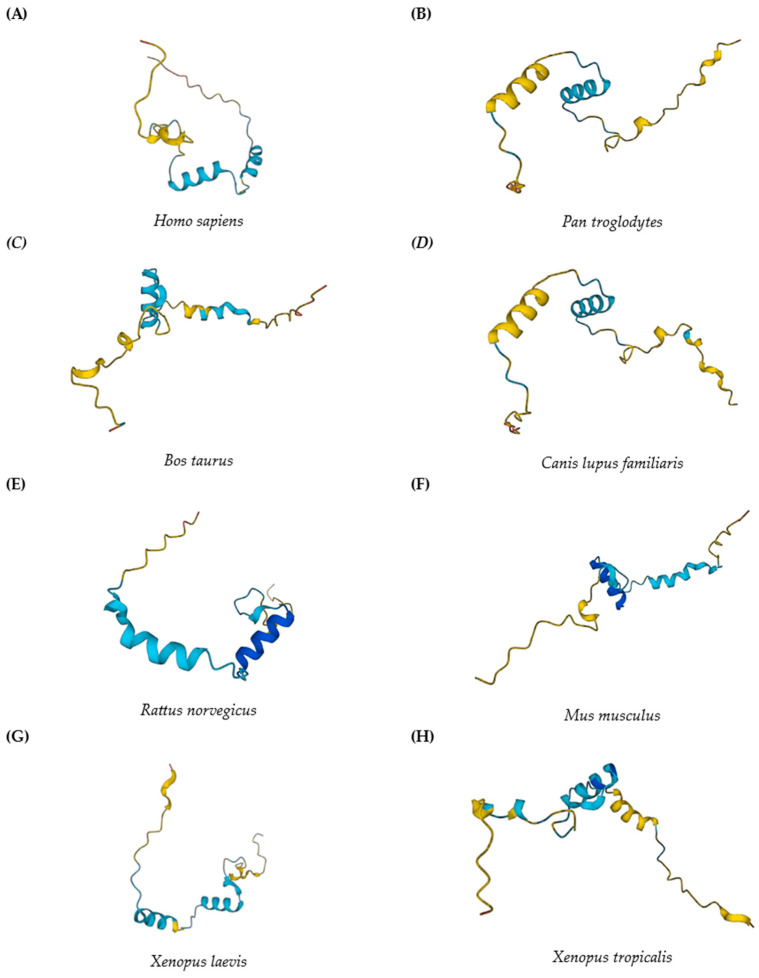
Molecular modeling of *Mustn1* in 3D AlphaFold. Protein structure predictions for *Mustn1* in various vertebrate organisms: (**A**) *H. sapiens*, (**B**) *P. troglodytes*, (**C**) *B. taurus*, (**D**) *C. lupus familiaris*, (**E**) *R. norvegicus*, (**F**) *M. musculus*, (**G**) *X. laevis*, and (**H**) *X. tropicalis*. All images are aligned to xy-axis. AlphaFold produces a per-residue model confidence score (pLDDT) between 0 and 100 and colored regions indicate pLDDT with Dark blue = Very high (pLDDT > 90); Light blue = High (90 > pLDDT > 70); and Yellow = Low (70 > pLDDT > 50). Predictions obtained from the AlphaFold Protein Structure Database (https://alphafold.ebi.ac.uk/ (accessed on June 1, 2024).

Musculoskeletal Temporarily Activated Novel Gene 1 (MUSTANG), also known as musculoskeletal embryonic nuclear protein 1 (*Mustn1),* was initially identified as an upregulated gene during fracture repair [11]. The *Mustn1* gene has three exons with two introns between them, codes for a protein consisting of 82 amino acids [12], and is known to be only expressed in vertebrate organisms, with structural homology between mammals (Figure 2). Initial investigations revealed that it is primarily expressed in adult skeletal muscle and tendon and, to a lesser extent, in bone and cartilage [11], which makes it a pan-musculoskeletal cell marker. The coded protein is the only known pan-musculoskeletal cell marker to date and does not belong to any recognized class of proteins [12].

**Figure 3:**
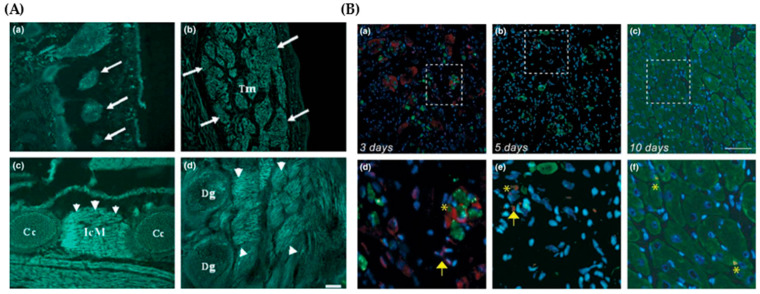
(**A**) *Mustn1 ^PRO^*-driven GFP expression at various stages of embryonic development in transgenic mice in (**a**) somites (white arrows), (**b**) trapezius muscle (what arrows), (**c**) intercostal muscles (white arrowheads), and (**d**) forelimb muscles (white arrowheads). (**B**) The study extended its observations to the context of skeletal muscle regeneration and repair following injury, showing a clear surge in *Mustn1*-GFP expression 3 days post injury, (**a**,**d**) with some areas overlaying with desmin (denoted by *) but numerous examples of mononuclear cells expressing only GFP (denoted by arrow). By 5 days post-injury (**b**,**e**) expression gradually subsided as newly formed muscle fibers (*) became more prominent, as shown by co-staining with GFP (red, arrow), DAPI (blue), and desmin (green). Finally, Desmin expression is most robust at 10 days post-injury (**c**,**f**), and only regions of GFP are evident overlay with desmin (*) and located at the periphery of newly formed muscle fibers. Tm, Trapezius muscle; IcM, Intercostal muscle; Dg, Digit; Cc, Costal cartilage. Modified from [14].

The promoter region of *Mustn1* was also isolated and characterized, revealing that its expression is influenced by several transcription factors, including AP-1 family members such as c-Fos, Fra-2, and JunD [13]. This isolation and characterization of the *Mustn1* promoter also facilitated the generation of *Mustn1^PRO^*-GFP (Green Fluorescent Protein) transgenic mice, revealing GFP expression in developing skeletal muscles, activated satellite cells, and during regeneration (Figure 3), thus providing an additional tool for studying *Mustn1* expression [14]. Suarez-Bregua et al. [15] also reported on the isolation and characterization of the zebrafish *mustn1b* promoter and revealed that the MyoD binding site was crucial for *mustn1b* expression in skeletal muscles. The same study also used this promoter to drive eGFP expression and detected fluorescence in the skeletal muscle pioneer cells and somites of embryos, in the craniofacial and fin muscles in larvae, as well as in the jaw, cranial muscles, tongue, heart, esophagus, and supracarinalis anterior, lateralis superficialis, and hypoaxial muscles in adult fish [15].

Additional studies from our laboratory have illustrated that *Mustn1* is primarily and selectively expressed within the musculoskeletal system [13,16,17,18]. Also, an array of comprehensive studies across various species, such as zebrafish [19,20], trout [21], ducks [22,23,24], chickens [25,26,27,28], pigs [29], donkeys [30], minks [31], and humans [32,33], has demonstrated *Mustn1* expression in various skeletal muscles, further implicating a role in this tissue. *Mustn1* was also showed to be involved in the myogenic differentiation of C2C12 cells, commonly used as a model system for muscle development [17].

*Mustn1* is also highly implicated in the development and regeneration of bone tissue. Its expression is prominently observed during early bone development and during the fracture repair process in periosteal osteoprogenitors, chondrocytes, and osteoblasts, highlighting its role in the complex cellular interactions necessary for effective bone regeneration [11,12,34,35]. Such dynamic expression underscores its potential importance in both normal bone healing and pathological conditions where bone repair processes may be disrupted [36]. In cartilage, *Mustn1* expression has been detected at various developmental stages and in response to mechanical stress, which is critical for maintaining the integrity and function of cartilage [11,16,18,20,37]. As *Mustn1* plays a significant role in normal cartilage formation and is also differentially expressed in pathological conditions like osteoarthritis, it appears that it is involved in chondrogenesis and in cartilage disease states. For example, *Mustn1* is upregulated in chondrocytes within the proliferative zones of the growth plate and articulate cartilage, which indicates its participation in the active growth phases of cartilage that are essential for proper joint function [35].

**Figure 4:**
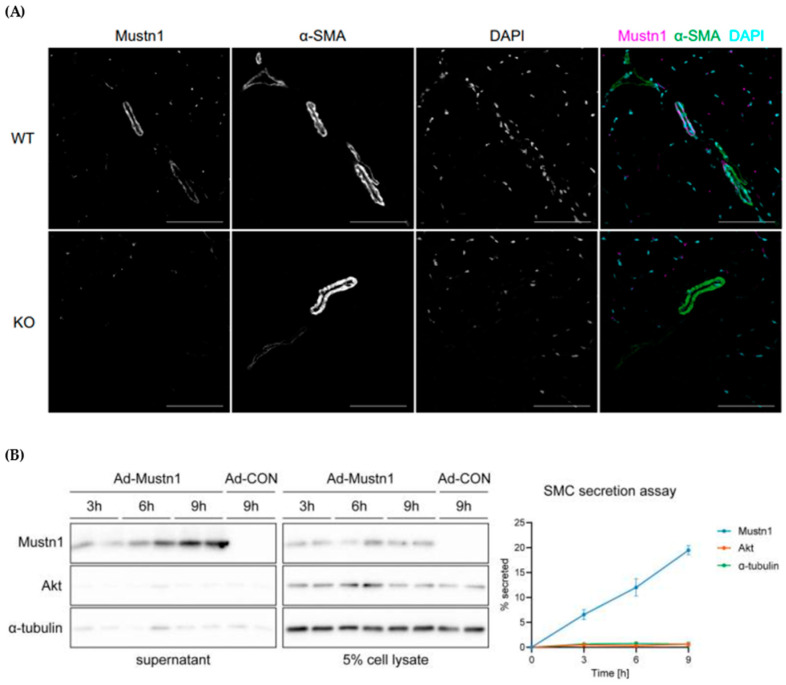
*Mustn1* expression in smooth muscle and as a secretory protein. (**A**) Immunofluorescence images of *Mustn1* co-expression with α-smooth muscle actin (α-SMA) in smooth muscle of blood vessels in tibialis anterior muscle cross sections from *Mustn1* knockout mice and wild-type littermates, scale bars: 100 μm (*n* = 3), (**B**) immunoblotting of cell supernatant and 5% cell lysate of cultured smooth muscle cells transduced with *Mustn1*-expressing adenovirus (Ad-*Mustn1*) and control adenovirus (Ad-CON), collected 3, 6, or 9 h after medium change, which shows increased *Mustn1* expression in cell supernatant. Representative blots (left panel) and quantification (right panel, *n* = 3). Data represented by mean ± SEM. Modified from [39].

*Mustn1* is also expressed in tendons [11], specifically, in tenocytes, particularly during phases of growth and repair, which reflects its role in tendon biology [38]. The expression of *Mustn1* in tendons suggests it may influence crucial processes such as tenocyte proliferation and the synthesis of extracellular matrix components, which are vital for tendon strength. More recently, Ducommun et al. [39] reported that *Mustn1* is also expressed in smooth muscle and suggested that it is a secreted microprotein that may play a role in the extracellular matrix (Figure 4). The notion of *Mustn1* as a secretory protein, in addition to a nuclear one, opens up additional potential avenues for explaining its broad impact on the various tissues of the musculoskeletal system.

## 3. Clinical Involvement

Beyond its structural and mechanical roles, skeletal muscle is intrinsically connected to various systemic physiological processes, including blood glucose regulation and bone health [40,41]. Moreover, it acts as an endocrine and paracrine gland [42], making the study of its development and function vital for multiple areas of biology and even medicine. When the development or function of skeletal muscle is dysregulated, pathologic conditions may ensue, including a group of more than 30 diseases characterized by muscle wasting [43]. These debilitating conditions involve mutations in genes vital for maintaining muscle integrity, which can result in either the dysfunction or the absence of essential muscle-specific proteins. Consequently, this leads to the progressive degeneration and weakening of the muscles, culminating in multiple forms of muscular dystrophy that impact different muscle fiber types [43,44].

Muscular dystrophies are a heterogenous collection of inherited disorders due to mutations in more than 40 genes, leading to the progressive degeneration and weakening of muscle [45]. The term muscular dystrophy encompasses a spectrum of disorders, including Duchenne, Becker, congenital, myotonic, Emery–Dreifuss, facioscapulohumeral, oculopharyngeal, and limb–girdle muscular dystrophies [46,47,48]. Each type of muscular dystrophy manifests with differing levels of severity, age of onset, inheritance patterns, and impacts on various muscle groups and even other organs [49,50].

The multiple symptoms of muscular dystrophies include, but are not limited to, muscle weakness and atrophy, reduced mobility due to joint stiffness, and respiratory complications, all negatively impacting patient quality of life and imposing significant burdens on patients and their families. Further, complications from muscular dystrophy extend to disruptions in the cardiac conduction system, leading to potentially life-threatening conditions such as fainting and sudden death. Additional symptoms may include facial weakness, pain, and swallowing difficulties [47]. For example, in the case of myotonic dystrophy, the clinical presentation is not limited to muscle weakness and myotonia but extends to nearly every system, including the endocrine one [51]. The manifestation of these symptoms typically hinders physical activities, including walking and daily functions, subsequently diminishing the quality of life and imposing substantial stress on both the patients and their families [52,53,54].

The current treatment options for muscular dystrophies include pharmacological, supportive, and gene and stem cell therapies [55]. Corticosteroids are widely used to improve muscle strength and delay the progression of the disease, especially for DMD [56,57]. There are drugs such as exon-skipping drugs and ataluren that facilitate the production of partially functional or functional dystrophin, respectively, in conditions with specific mutations [58,59,60]. Emerging treatments include gene therapy, such as CRISPR, to correct gene mutations and stem cell therapy that focuses on regenerating the damaged muscle tissue [61,62]. There are other experimental drugs that are under development [55]. Supportive therapies include physiotherapy, occupational therapy, and nutritional support to maintain muscle function and to manage complications in advanced stages [56].

**Figure 5:**
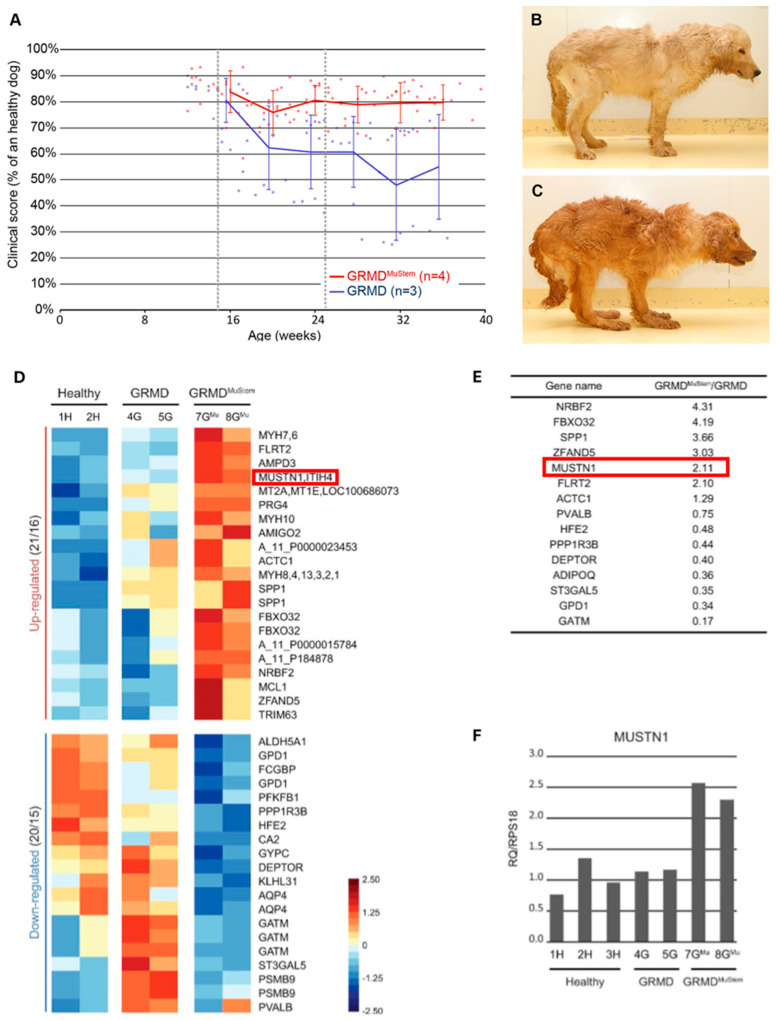
*Mustn1* in Golden Retriever Muscular Dystrophy (GRMD) dogs treated with stem cells from the hindlimb muscle and assessed weekly with (**A**) clinical score as a percentage of a theoretical healthy dog score, (**B**) stem-cell treated dogs (GRMD^MuStem^) show a plateau in their score, (**C**) untreated dogs (GRMD) show a continued decline in their score. PCR performed on the bicep femoris showing that (**D**) *Mustn1* is one of the regulatory genes that were upregulated in GRMD^MuStem^ dogs and (**E**) one of the top 5 upregulated genes, (**F**) compared to healthy and GRMD dogs, *Mustn1* was significantly upregulated in GRMD^MuStem^ dogs. Modified from [64].

Although there is no clear known connection, the relationship between *Mustn1* and muscular dystrophies is plausible due to the protein’s significant role in muscle development and repair. Since muscular dystrophies are characterized by the progressive weakening and loss of muscle mass, it is conceivable that they may be influenced by the expression and function of *Mustn1*, which is known to be associated with muscle regeneration [14,39]. *Mustn1* is also notably associated with the differentiation and fusion of muscle cells, processes fundamental to the formation and maintenance of healthy muscle tissue [17,28]. As such, disruption or alteration in *Mustn1* expression or function could potentially exacerbate the symptoms of muscular dystrophies or contribute to the onset of these conditions. Indirect evidence for this comes from Balagopal et al. [63], who reported that Duchenne Muscular Dystrophy (DMD) involves an imbalance between the rates of muscle protein synthesis and degradation. Treatment with an anabolic steroid, oxandrolone, enhanced MHC synthesis, subsequently reducing muscle degeneration. Interestingly, in response to oxandrolone, *Mustn1* was among the genes that were downregulated by more than twofold in DMD muscles. Robriquet et al. [64] demonstrated the clinical benefits of transplanting skeletal muscle-resident stem cells into dogs with Golden Retriever Muscular Dystrophy (GRMD), a condition that exhibits signs and symptoms akin to those of DMD, the most prevalent muscular dystrophy in humans [65]. This transplantation led to an enhancement in skeletal muscle fiber regeneration, with *Mustn1* being one of the genes prominently upregulated during the regenerative phase (Figure 5).

Kennedy’s Disease/Spinobulbar Muscular Atrophy is characterized as an X-linked, androgen-dependent neuromuscular disease, fundamentally resulting from mutations in the androgen receptor gene [66,67]. This condition presents with progressive weakness of proximal limbs and bulbar muscles, alongside androgen insensitivity symptoms such as gynecomastia and infertility and additional sensory deficits. It was originally and predominantly categorized as a “motor neuron disease” but it may have myogenic origins [68,69]. A study focused on transcriptional alterations in muscles with overexpressed androgen receptor and reported significant motor dysfunction and the dysregulation of several genes, including *Mustn1*, which was upregulated in both male and female muscles, suggesting its involvement in muscle recovery post androgen withdrawal and as a potential compensatory mechanism against muscle deterioration [70].

Understanding the molecular mechanisms and interactions of *Mustn1* with other proteins and pathways implicated in muscular dystrophies will reveal new insights into the pathological mechanisms of these conditions. Furthermore, according to Matre et al. [71], accumulating evidence suggests that DMD may be a stem cell disease, and restoring dystrophin via CRISPR/Cas9 resulted in significant upregulation of Pax7 satellite cells. These insights highlight the critical role that individual genes such as Pax7 and/or possibly *Mustn1* can play in satellite cells and in skeletal muscle regulation.

## 4. *Mustn1* in Skeletal Muscle

Diverse research experiments have also demonstrated the upregulation of *Mustn1* expression during muscle exercise and hypertrophy, indicating its possible role in muscle plasticity and adaptive responses to mechanical load. In studies examining the impact of physical activity on *Mustn1* expression, McKenzie et al. [72] observed that an acute aerobic run led to the upregulation of *Mustn1* in the soleus muscle of rats. Similarly, Jensen et al. [73] reported increased *Mustn1* expression in the bicep femoris muscle of pigs during the early recovery phase post acute physical activity. Additionally, resistance training has been linked to elevated *Mustn1* expression in human quadricep muscles following muscle lengthening and shortening [32]. In rats, *Mustn1* expression was also upregulated in the flexor halucis longus muscle after climbing a ladder with weights tied to the tail [74,75], and in mice, in gastrocnemius muscles after unloading and reloading of the hindlimbs [39].

**Figure 6:**
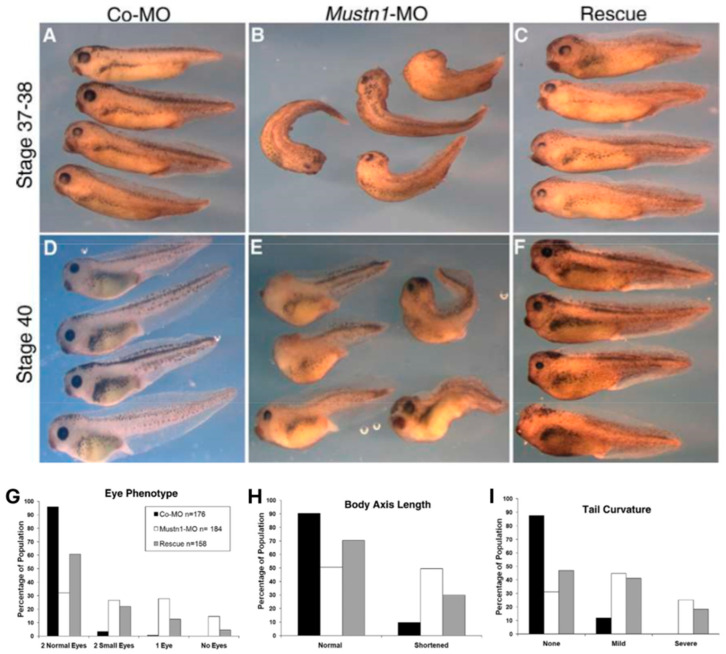
*Mustn1* perturbation in *Xenopus laevis*. Antisense *Mustn1* MO injected at the 4-cell stage targeting the start codon of *Mustn1* mRNA led to distinct malformations of the eye, body axis length and tail curvature (**A**,**B**,**D**,**E**). Rescue group with *Mustn1*-MO embryos co-injected with a modified, MO-resistant *Mustn1* mRNA, significantly reduced or corrected the developmental defects observed (**C**,**F**). Quantitative analysis of (**G**) eye, (**H**) body axis length, and (**I**) tail curvature. Modified from [18].

Functionally, *Mustn1* appears to be integral to myotube formation and muscle cell differentiation. Silencing of *Mustn1* via RNA interference (RNAi) resulted in impaired myogenic differentiation and the downregulation of key fusion markers, leading to suppressed myotube formation [17]. More strikingly, when antisense morpholino oligonucleotides (MOs) targeted the start codon of *Mustn1* mRNA, its downregulation led to developmental abnormalities in *Xenopus*, affecting the eye, body axis length, and tail curvature [18] (Figure 6). In zebra fish, antisense MOs to knockdown *mustn1a* as one of Foxj1-induced genes, resulted in multiple ciliary dysfunction-associated phenotypes such as curved body axis, otolith defects, left–right asymmetry abnormalities, curling of cilia, and disorganized γ-tubulin expression during ciliogenesis and cilia organization. [19]. Further, *Mustn1* is dynamically expressed in activated mouse Pax7-positive skeletal muscle satellite cells across various stages of embryonic development, but its natural expression peaks at 3 months of age [17], as well as during phases of skeletal muscle repair and regeneration [14]. More recently, further investigation by Hu et al. [76] examined the effect of *Mustn1* on the proliferation of skeletal muscle satellite cells isolated from the chicken pectoralis muscle. When *Mustn1* was silenced using small interfering RNA (siRNA), a notable decrease was observed not only in the relative expression of the Pax7 satellite cell marker, but also in the actual satellite cell count and proliferation. Conversely, overexpression of *Mustn1* led to a significant increase in both Pax7 expression and satellite cell proliferation. These findings (Table 1) collectively underscore that *Mustn1* is an important regulatory protein for skeletal muscle, especially in the proliferation and differentiation of skeletal muscle satellite cells.

However, we recently reported that ablation of *Mustn1* in skeletal muscle resulted in no major phenotypic changes in the organism or in individual muscles, with the exception of a temporal lower weight up to 3 months [77]. This suggest that ablation of *Mustn1* does not severely impair the development of skeletal muscle. These findings stand in contrast to prior research that showed developmental abnormalities in *Xenopus* [18] and inhibitory effects on C2C12 myofusion and myotube formation [17], as well as on satellite cells [76]. While the RNAi-mediated silencing of *Mustn1* significantly reduced the expression of all myogenic differentiation and fusion markers in vitro, the in vivo study did not show any major or significant changes [77]. This may be due to the different approaches employed in these various studies. But investigations into the consequences of *Mustn1* ablation in Pax7-positive skeletal muscle satellite cells [64,65] did identify dynamic shifts in physiological effects in knockout (KO) vs. wild-type (WT) mice (age 2 to 4 months). These shifts manifested as variations in effect significance: while certain effects were pronounced at 2 months, they might not be so at 4 months, and vice versa (Table 2).

Notably, these changes include a statistically significant temporal reduction in body weight, alterations in glucose tolerance facilitated by variations in the expression of GLUT channels and metabolism-related genes such as MUP-1, and a decrease in OSTN [77]. Concurrently, adjustments in locomotor dynamics are evident from changes in grip strength and vertical ground force during hindlimb gait. Additionally, the significant alterations in the ex vivo contractile properties and fiber composition of the soleus muscle underscore the wide impact of *Mustn1* ablation on skeletal muscle, while no significant differences were observed in extensor digitorum longus (EDL) muscles. Given that the soleus, a major plantar flexor muscle, is essential for activities such as walking, running, and climbing, the observed increase in Type IIb fibers may contribute to several notable changes. These include the elevated single limb vertical force during walking and greater absolute contractile forces in the KO mice during the ex vivo isometric contraction tests [78].

Minchew et al. [79] reported that various mouse strains display distinct muscle fiber composition, which affects isometric contractile force and fatigue test outcomes, suggesting that the differences observed in our study may be attributed to varied fiber compositions in the soleus muscle. This discrepancy may be related to the role of *Mustn1* in primarily regulating Type I myofiber development during myogenesis, with Type II fibers appearing later [2]. Past research showed that alterations in specific genes can precipitate changes in muscle fiber types, often resulting in increases in Type IIb fibers [80,81,82,83,84]. These investigations underscore the role that certain genes play in modulating fiber composition within skeletal muscle, with the consequence of traditionally oxidative muscle types switching to adopt more glycolytic characteristics. Further, certain genes were found to induce skeletal muscle hypertrophy and hyperplasia at the same time [80].

A distinct experiment [39] utilizing *Mustn1* KO-first and MEF2C/myogenin promoter–Cre recombinase crossed with β-actin promoter-driven Flp recombinase or Actb-Cre mice to ablate *Mustn1* showed a different outcome compared to the Pax7-Cre mediated gene deletion. In this study, no significant changes were observed in body weight, ex vivo contractile forces, or glucose tolerance. However, this study indicated different responses of skeletal muscles in various experiments (Table 3). Interestingly, this approach also revealed temporal differences, with observed changes in gene expression only in 8-week-old mice, but not in 3–4-month-old mice [39].

While the precise mechanism(s) underlying *Mustn1* role in myogenesis is not fully understood, emerging evidence suggests it has a more pronounced impact on Type I muscle fibers. For instance, McKenzie et al. [72] demonstrated that aerobic exercise specifically enhances *Mustn1* expression in the soleus muscle, which predominantly consists of Type I fibers, unlike the gastrocnemius muscles that contain a mix of both Type I and II fibers. Supporting this observation, Ducommun et al. [39] reported variable *Mustn1* responses across different muscles to the same exercise modalities: uphill treadmill running led to a significant ~three-fold increase in *Mustn1* expression in the soleus but not in the gastrocnemius or tibialis anterior, another muscle with mixed fiber types; downhill treadmill running resulted in a ~1.5-fold increase in *Mustn1* expression in the gastrocnemius and a ~three-fold increase in the soleus, with no notable change in the tibialis anterior; and free-wheel running increased *Mustn1* expression by ~1.5-fold only in the soleus. These findings collectively suggest a potential role of *Mustn1* in adapting Type I muscle fibers to physical activity.

There is a potential specific relationship between *Mustn1* and Type I fibers that may explain their reduction observed at both 2 and 4 months in the Pax7-Cre-mediated *Mustn1* KO mice [78]. Further, considering that Type II fibers develop from Type I fibers [2], the decrease in the regional area percentage of Type IIa fibers could be a downstream effect of the diminished number of Type I fibers. Yet, the role of *Mustn1* in the maturation or transition of these muscle fibers from Type I remains speculative. Interestingly, *Mustn1* KO mice generated Type IIb fibers when their WT counterparts did not [78], hinting at a possible compensatory mechanism to offset lower Type I fiber counts. However, the question remains why a Type I fiber would evolve into a Type IIb instead of a Type IIa fiber, providing an interesting direction for future research.

Ablation of *Mustn1* in the skeletal muscle of mice significantly altered gene expression, with 213 genes upregulated and 93 downregulated, suggesting extensive interconnections with other genes within the tissue [77]. This indicates a potential complex regulatory network, possibly influencing various molecular processes of skeletal muscle development and function. Genes such as FHL2, FGFR2, HS6ST2, CSRP3, INCENP, and NDE1, along with *Mustn1*, have been associated with muscle growth, satellite cell proliferation, and muscle hypertrophy in broiler and layer chickens [85]. Additionally, MYTHO (Macroautophagy and Youth Optimizer), a regulator of autophagy and skeletal muscle integrity, plays a significant role in various models of skeletal muscle atrophy. Its short-term depletion in mice mitigates muscle atrophy caused by conditions such as fasting, denervation, cancer cachexia, and sepsis, while its overexpression induces muscle atrophy. Intriguingly, the knockdown of MYTHO leads to a progressive increase in muscle mass, accompanied by the upregulation of *Mustn1* [86].

The dynamic expression of *Mustn1* in skeletal muscle satellite cells plays a role in regulating muscle development, repair, and regeneration. Its intricate involvement in modulating gene expression underscores the complexity of its functions across various physiological and pathological conditions. Collectively, these data demonstrate that *Mustn1,* not only influences muscle cell proliferation and differentiation, but also impacts how skeletal muscle responds to physical stress and disease states. The varied responses to *Mustn1* ablation, from shifts in gene expression to alterations in muscle fiber characteristics, highlight the gene’s significance in muscle’s genetic networks, signaling pathways, as well as physiology and pathophysiology.

## 5. Future Research Directions

Given the broad implications of these findings, further research is essential to unravel the precise molecular mechanisms by which *Mustn1* is involved in diverse processes. Such studies could lead to targeted therapies that modulate *Mustn1* activity to treat or manage skeletal muscle-related diseases, enhance muscle repair, or potentially modulate muscle aging. Future investigations should also explore the interaction of *Mustn1* with other proteins and molecular pathways, enhancing our understanding of muscle biology, and pave the way for novel interventions in muscle-related disorders. Understanding the molecular mechanisms and interactions of *Mustn1* with other proteins and pathways implicated in muscular dystrophies can reveal new insights into the pathological mechanisms of these conditions. Exploring these interactions could potentially identify new targets for therapeutic intervention and provide a foundation for the development of innovative therapeutic strategies. Therefore, investigating the fundamental molecular processes of myogenesis, involving potential regulatory genes such as *Mustn1*, is significant for devising future comprehensive strategies to prevent and treat skeletal muscle wasting (e.g., diabetes, sarcopenia, dystrophies) and injuries arising from direct (e.g., lacerations, contusions, and strains) and indirect (e.g., ischemia and neurological dysfunction) causes [87,88].

To attain a more intricate understanding of *Mustn1* role and function in skeletal muscle, it is pivotal that future research endeavors probe into areas unexplored in the aforementioned studies. One area for future inquiry is a detailed examination of the repercussions of *Mustn1* ablation on muscle repair and regeneration. Afterall, *Mustn1* is upregulated during skeletal muscle repair [14,39]. Investigating the implications and subsequent effects of *Mustn1* ablation on the muscle repair process in much greater detail than simply focusing on its expression constitutes a logical approach. This aspect is fundamental, as it provides an opportunity to understand the intricacies of how *Mustn1* ablation can influence the biological processes that underlie muscle repair. It has the potential to unravel the nuanced roles of *Mustn1* in maintaining muscle structural and functional integrity post-injury. Moreover, analyzing the potential influence of exercise on muscles in the absence of *Mustn1* is crucial to understand the modulation of physiological responses to physical activity by this protein. Further scrutiny of its impact on muscle groups, especially those abundant in Type I fibers, is essential to ascertain whether its influence is homogenous across varying muscle types or exhibits diversified effects contingent on the specific muscle group studied.

Given the pan-musculoskeletal marker nature of *Mustn1*, the ramifications of its function extend beyond skeletal muscle, encompassing diverse aspects such as weight and glucose tolerance [77]. *Mustn1*’s effects in various aspects of skeletal muscle function [78] necessitates a deeper, more focused examination of its molecular functions and the regulatory pathways it is involved in. This exploration could provide vast, diversified insights, significantly contributing to the understanding of skeletal muscle metabolism and physiopathology.

In the realm of metabolic processes, an exploration of the relationship between insulin, mitochondria, and *Mustn1* is of paramount importance to elucidate the complex interactions within the body’s metabolic machinery. A meticulous, focused investigation is imperative to dissect the intricate metabolic interrelations, aiming to provide an in-depth understanding of the mechanisms through which *Mustn1* may modulate mitochondrial biogenesis and/or activities in relation to insulin and GLUT proteins and, subsequently, influence the pathways governing glucose metabolism within the physiological system.

Given the crucial role of insulin in glucose homeostasis and considering the key function of GLUT proteins in facilitating glucose transport across cellular membranes, understanding the nuanced interactions between *Mustn1* and insulin can offer valuable insights into the regulatory mechanisms of glucose metabolism. Such exploration can illuminate how changes in *Mustn1* expression or function can potentially impact insulin, mitochondria, and the expression of GLUT proteins, thereby influencing glucose uptake in skeletal muscle, and will require biochemical assays, molecular biology techniques, and metabolic studies. Understanding the potential alterations in cell signaling cascades, the changes in GLUT protein expression, and the modifications in metabolic responses due to *Mustn1* levels could provide insights into the physiological and pathological implications of *Mustn1* in metabolic processes. Comprehensively analyzing the molecular interactions and biochemical pathways involving *Mustn1*, mitochondria, insulin, and GLUT proteins, could also unravel the precise mechanisms and consequences of their interplay. Such advancements in knowledge of the metabolic implications of *Mustn1* can contribute to the broader understanding of metabolic physiology and pathophysiology, paving the way for innovations in the treatment of not only skeletal muscle diseases, but also metabolic diseases and enhancing our ability to manipulate such processes therapeutically.

The differential effects of aging on slow and fast muscle fibers underscore a complex interplay of metabolic processes and protein quality control mechanisms that may be further influenced by changes in fiber type distribution, such as those observed following *Mustn1* ablation [78]. Research by Murgia et al. [89] highlights that, while the mitochondrial content declines in both fiber types with age, glycolysis and glycogen metabolism exhibit divergent patterns, being upregulated in slow fibers and downregulated in fast fibers. Additionally, aging mitochondria show decreased expression of the redox enzyme monoamine oxidase A. In terms of protein quality control, slow fibers upregulate a subset of actin and myosin chaperones, which contrasts with the downregulation observed in fast fibers. These metabolic and sarcomeric adaptations are critical, as they relate to the observed capacity of slow fibers to maintain their mass during aging, unlike fast fibers. Investigating the impact of *Mustn1* on fiber-specific aging should provide deeper insights into its role in muscle aging and the potential for targeted interventions to mitigate age-related muscle deficits.

The persistence of residual *Mustn1* expression in skeletal muscle despite its ablation has raised pivotal questions regarding its origins [77]. Although the residual expression of *Mustn1* might be attributed to the inclusion of tendons or vascular smooth muscle within the skeletal muscle samples, particularly in light of recent findings that indicate *Mustn1* expression in smooth muscle cells [39], such minimal expression may be indicative of potential downstream events, possibly manifesting elevated *Mustn1* expression in alternate cell types such as fibroblasts or chondroblasts, which can be hypothesized as a compensatory mechanism addressing the incurred loss of *Mustn1* in skeletal myocytes. Thus, an extensive exploration of molecular data across diverse cell populations via single-cell transcriptomics at early developmental phases will be necessary to elucidate the presence of *Mustn1* in the various cell lineages.

Given that *Mustn1* is expressed across the primary tissues in the musculoskeletal system, specifically in bone, cartilage, and skeletal muscle [8,9], developing new conditional KO mice models that incorporate double- and/or triple-tissue *Mustn1* deletion would enable a more comprehensive exploration of its role within the entire musculoskeletal system. Previous research illustrating the transformative potential of fibroblasts to myoblasts in the presence of myoD [90,91,92] further points to the dynamics of cell interactions. Analyzing the consequences of *Mustn1* ablation across multiple tissue combinations within the musculoskeletal system will enhance our knowledge of its varied roles and thus broaden our comprehension of musculoskeletal physiology and pathology.

Moreover, it would be interesting to also explore the expression and possible involvement of *Mustn1* in various bone states, beyond fracture repair. For example, examination of *Mustn1* expression during osteopenia and osteoporosis may reveal insights into whether its expression can be regulated by mechanical loading/unloading. If indeed its expression is altered in osteopenic and osteoporotic bones, this may enable us to use it as a potential marker for these disease states, especially in its secreted form, as was recently proposed [39].

Ducommun et al. [39] proposed that in addition to being a nuclear protein, *Mustn1* is secreted from smooth muscle, which makes this an interesting and novel observation. Therefore, additional experiments with skeletal myocytes, chondrocytes, or osteoblasts and, more importantly, deciphering *Mustn1* role as an extracellular microprotein are all warranted. In the context of *Mustn1* as an ECM protein, Ducommun et al. [39] speculated that it is possible that its receptor exists in target cells and that *Mustn1* can remain associated with the ECM to modulate the activity of smooth muscle cell receptors. Obviously, this remains to be experimentally determined, as is *Mustn1* role in fibro–adipogenic progenitors (FAPs) and pericytes (which also express *Mustn1*), especially as it relates to ECM remodeling.

To validate that *Mustn1* functions as a key signaling molecule within the musculoskeletal system, further detailed molecular studies are essential. These studies should focus on delineating the expression patterns of *Mustn1* in conjunction with other regulatory genes. With over 300 genes impacted by *Mustn1* ablation in skeletal muscle, identifying the sequential expression of these genes and understanding their genetic interactions will help clarify *Mustn1* role in musculoskeletal genetic networks. This could potentially lead to novel insights into its regulatory mechanisms and physiological effects. For example, exploring the interactions and influence of genes such as ccndbp-1, a positive skeletal myogenic differentiation regulator, and *Mustn1* on muscle development will offer a broader context of muscle physiology and aging. The known association of ccndbp-1 with MyoD [93] provides a common key differentiation factor for investigating *Mustn1* interaction with or influence on other critical regulatory proteins within skeletal muscle during various stages of muscle development or regeneration.

Additionally, considering the age-related changes in muscle function as detailed by Hill et al. [94], which highlight variations in the soleus and EDL muscles’ power and fatigue resistance with age, investigating *Mustn1* in the context of aging could determine if it has a modulatory effect on muscle quality, particularly in eccentric and concentric muscle functions. Understanding this relationship might also provide insights into the differential impacts of aging on different muscle types and how *Mustn1* could be involved in age-related declines in muscle function.

Future research investigating the interactions between *Mustn1* and other key regulators of satellite cell function is also a worthy pursue. For example, it would be valuable to examine whether *Mustn1*, as a nuclear protein, engages with epigenetic factors such as Prmt5, a regulator that controls the proliferation of adult skeletal muscle stem cells without affecting their initial activation [95]. This exploration is particularly relevant because both Prmt5 and *Mustn1* influence satellite cell proliferation, with *Mustn1* notably inactive when satellite cells are in a quiescent state [14].

The ensuing questions and unexplored research areas identified by the aforementioned findings by our laboratory and others underscore the need for future experiments designed to decipher the multifaceted roles and influence of *Mustn1* in musculoskeletal physiology and pathology, potentially revealing novel insights into cell interactions, differentiation, and other molecular mechanisms. Recent research by Ducommun et al. [39] adds a new dimension to *Mustn1* function, showing that it is also expressed in smooth muscle. Elucidating the exact molecular function(s) of *Mustn1* will enhance our fundamental understanding of this very interesting gene/protein and potentially lead to the discovery of novel therapeutic targets and strategies for addressing a multitude of skeletal muscle conditions.

## Figures and Tables

**Table 1:** Summary of *Mustn1* functional perturbation studies.

Cell/Tissue	Approach	Observed Effects	Study
Myogenic cells (C212)	RNAi	Impaired myoblast differentiation, myofusion, and myotube formation with downregulation of differentiation and fusion factors	[17]
Frog Embryo	Antisense MO	Small or absent eyes, shortened body axis, and tail curvature	[18]
Zebrafish embryo	Antisense MO	Curved body axis phenotype; otolith and left–right asymmetry defects Curling of cilia and disorganized γ-tubulin expression	[19]
Chicken Pax7 satellite cells	siRNA	Decreased relative expression, proliferation, differentiation, and myotube formation with downregulation of differentiation factors	[76]

**Table 2:** Summary of *Mustn1* functional perturbation studies. Summary of significant differences observed after *Mustn1* ablation in skeletal muscle of WT and KO mice within the same age groups at 2 and 4 months. MUP-1 = major urinary protein 1; Scl25a10 = mitochondrial solute carrier protein; OSTN = osteocrin; PCSA = physiological cross-sectional area (**** = *p* < 0.0001; *** = *p* < 0.001; ** = *p* < 0.01; * = *p* < 0.05; ns = non-significant).

Experiments	2 Mo KO	4 Mo KO
**Weight**	Decreased **	ns
**Glucose Metabolism**	Higher tolerance ****	ns
**Metabolism-related genes**	MUP-1 ***, Scl25a10 *, OSTN *	MUP-1 *
**Grip Strength (absolute)**	Decreased **	ns
**Single Limb Force**	ns	Increased hindlimb vertical *
**Ex vivo: absolute force (soleus)**	Increased 100 **, 150 ***, 300 *** Hz	Decreased 20 ** Hz
**Ex vivo: specific force (soleus)**	Decreased at 20 * Hz	Decreased 20 ***, 60 ** Hz
**Ex vivo: fatigue (soleus)**	Higher ****	Higher *
**PCSA (soleus)**	ns	Increased *
**Muscle fiber composition (soleus)**	ns	Decreased Type I * Decreased Type IIa * Increased Type IIb *

**Table 3:** Observed effects in various experiments in skeletal muscle on *Mustn1* expression and function compared to the control group (**** = *p* < 0.0001; *** = *p* < 0.001; ** = *p* < 0.01; * = *p* < 0.05; ns = non-significant). Data from [39].

Experiments	Muscle	Observed Effects
Acute exercise (uphill treadmill)	Gastrocnemius	ns
	Soleus	*Mustn1* expression increased ****
	Tibialis anterior	ns
Acute exercise (downhill treadmill)	Gastrocnemius	*Mustn1* expression increased *
	Soleus	*Mustn1* expression increased *
	Tibialis anterior	ns
Exercise training (free-wheel running)	Gastrocnemius	ns
	Soleus	*Mustn1* expression increased *
	Tibialis anterior	ns
Hindlimb unloading/reloading	Gastrocnemius	*Mustn1* expression increased **** 1 day of hindlimb reloading
	Vasus lateralis (human)	*Mustn1* expression decreased ** after 10 days of unloading *Mustn1* expression increased ** after 21 days of active recovery
Muscle injury	Gastrocnemius (Bacl2 injection)	*Mustn1* expression increased **** 1 day post-injury
	Tibialis anterior (cardiotoxin)	Collagen content increased * in female *Mustn1* KO mice (not in males) 14 days after injury.
Muscular dystrophy model	EDL	*Mustn1* expression increased ***
	Psoas	*Mustn1* expression increased *
Femoral artery ligation	Tibialis anterior	*Mustn1* expression increased ** 14 days post-ligation.

## Data Availability

No new data were created or analyzed in this study. Data sharing is not applicable to this article.
